# Klotho Ameliorates Vascular Calcification via Promoting Autophagy

**DOI:** 10.1155/2022/7192507

**Published:** 2022-10-26

**Authors:** Lufeng Li, Wei Liu, Qi Mao, Denglu Zhou, Keqi Ai, Wei Zheng, Jihang Zhang, Lan Huang, Shangcheng Xu, Xiaohui Zhao

**Affiliations:** ^1^Institute of Cardiovascular Research, Xinqiao Hospital, Army Medical University, Chongqing 400037, China; ^2^Department of Cardiology, Guangzhou Institute of Cardiovascular Disease, Guangdong Key Laboratory of Vascular Diseases, State Key Laboratory of Respiratory Disease, The Second Affiliated Hospital, Guangzhou Medical University, Guangzhou 510260, China; ^3^Institute of Immunology, Army Medical University, Chongqing 400038, China; ^4^Department of Radiology, Xinqiao Hospital, Army Medical University, Chongqing 400037, China; ^5^Center of Laboratory Medicine, Chongqing Prevention and Treatment Center for Occupational Diseases, Chongqing 400060, China

## Abstract

Vascular calcification (VC) is regarded as a common feature of vascular aging. Klotho deficiency reportedly contributes to VC, which can be ameliorated by restoration of Klotho expression. However, the specific mechanisms involved remain unclear. Here, we investigated the role of autophagy in the process of Klotho-inhibiting VC. The clinical study results indicated that, based on Agatston score, serum Klotho level was negatively associated with aortic calcification. Then, Klotho-deficient mice exhibited aortic VC, which could be alleviated with the supplementation of Klotho protein. Moreover, autophagy increased in the aorta of Klotho-deficient mice and protected against VC. Finally, we found that Klotho ameliorated calcification by promoting autophagy both in the aorta of Klotho-deficient mice and in mouse vascular smooth muscle cells (MOVAS) under calcifying conditions. These findings indicate that Klotho deficiency induces increased autophagy to protect against VC and that Klotho expression further enhances autophagy to ameliorate calcification. This study is beneficial to exploring the underlying mechanisms of Klotho regulating VC, which has important guiding significance for future clinical studies in the treatment of VC.

## 1. Introduction

Vascular calcification (VC) is associated with increased risk of major adverse cardiovascular events in several clinical conditions, such as chronic kidney disease and atherosclerosis and aging [1, 2]. The formation of VC is associated with complex pathological mechanisms, including osteogenic differentiation and apoptosis of vascular smooth muscle cells (VSMCs) and release of matrix vesicles loaded calcium (Ca) and phosphate (Pi) [3, 4]. By inhibiting these processes, VC can be effectively treated.

Klotho, a protein highly expressed in the kidney, is thought to be involved in various aging-associated pathologies [5]. Studies have reported that Klotho-deficient mice developed obvious aortic VC, which can be reversed by Klotho overexpression [6, 7]. To date, the mechanisms whereby Klotho protects against VC have focused on not only its role as an obligate co-factor for FGF23 signaling in regulating Pi and vitamin D systems in kidney, but also its direct effects on the vasculature as a circulating anti-calcific factor [8–10]. However, the mechanisms of this direct effects have not yet been fully explored.

Growing evidence indicates that autophagy, defined as the dynamic, refined, and controlled process of cellular self-digestion, protects VSMCs against calcification [11, 12]. Although autophagic activity reportedly increases in the aorta of Klotho-deficient mice [13], its role in the Klotho's regulation of VC remains unclear. The present study investigated whether Klotho deficiency could induce protectively-increased autophagy and whether Klotho administration ameliorated calcification through said autophagy increase.

## 2. Materials and Methods

### 2.1. Clinical Study

The clinical study protocol complied with the Declaration of Helsinki and was approved by the Xinqiao Hospital Ethics Committee, Army Military Medical University (Chongqing, China). Twenty-seven randomly selected participants between the age of 55 and 85 were consecutively recruited from the Physical Examination Center of Xinqiao Hospital between May 2019 and September 2019. They were examined using 64-row multislice computed tomography (CT; Japan) to detect the extent of calcification of aorta (above the diaphragm). The Agatston score was used to assess severity of VC and was evaluated using the Agatston method [14]. Briefly, all image data acquired through chest CT or coronary angiography were analyzed using semiautomatic software (Syngo Calcium Scoring CT, Siemens, Germany) by experienced radiologists. The scores were separately calculated for three parts of aorta, namely, the ascending aorta, aortic arch, and thoracic aorta. Then, the total aortic calcification score was summed and recorded as Agatston score. The blood samples of the participants were collected and centrifuged at 300 rpm for 10 min at 4°C, and sera were stored at -80°C until further analysis. The serum Klotho level was measured using a Human Klotho ELISA Kit (CSB-E13235h, Wuhan Huamei Biotechnology Company, China).

### 2.2. Animal Study

Klotho homozygous (HZ) mice were obtained from Prof. Gu Jun at the State Key Laboratory of Protein and Plant Gene Research, College of Life Science, Peking University (Beijing, China). HZ mice were crossed to obtain Klotho wild-type (WT) and knockout (KO) mice. Each group employed four-week-old male mice in groups of three in the animal experiments. The animals were housed at the Animal Experimental Center, Xinqiao Hospital, Army Medical University. All animal protocols were approved by the Army Medical University Institutional Animal Care and Use Committee before preforming the study and conformed to the regulations of Guide for the Care and Use of Laboratory Animals (8^th^ edition, National Research Council, USA, 2011). After intraperitoneal anesthesia using 40 mg/kg of pentobarbital, the mice were sacrificed to obtain aorta.

KO mice and their WT littermates were administered 1.2 mg/kg/day rapamycin (RA; HY10219, MCE, China) or saline for 2 weeks, as previously described [15]. KO mice and their WT littermates were also administered 50 mg/kg/day chloroquine (CQ; C6626, Sigma, USA) or saline for 2 weeks, as previously described [16]. Furthermore, KO mice and their WT littermates received 0.02 mg/kg Klotho recombinant protein (KL; 5334-KL-025, R&D Systems, USA) or saline every 48 h for 2 weeks [17]. All treatments were administered through intraperitoneal injection.

### 2.3. Cell Culture

Mouse aortic vascular smooth muscle cells (MOVAS; ATCC ® CRL−2797, American Type Culture Collection, Manassas, VA, USA) retained a VSMC phenotype and were cultured in Dulbecco's modified Eagle's medium (DMEM; Gibco, USA) containing 10% fetal bovine serum (FBS; Gibco) and 1% penicillin-streptomycin (Beyotime, China) in a 5% CO_2_ incubator at 37°C. Only cells between passages two and five were used in the experiments. MOVAS cells were identified via immunofluorescence detection of *α*-SMA expression, as described previously [18].

MOVAS cells were treated with 0.01 mM RA for 24 h or 20 mM CQ for 4 h, as previously described [16, 19]. Further, cells were treated with 100 ng/mL KL for 15 min, as previously described [20].

### 2.4. Induction of Cellular Calcification

At 80% confluence, the cells were transferred to a calcification medium (CM) containing 5% FBS, 10 mM *β*-GP (Sigma), 0.25 mM L-ascorbic acid (Sigma), and 10^−8^ mM dexamethasone (Sigma), according to a previous report [21]. The CM was changed every other day.

### 2.5. Ca Staining and Content Analysis

Calcification in the mouse aorta was observed using Von Kossa staining kit (ab150687, Abcam, UK). Briefly, aortic tissue sections were incubated with 5% silver nitrate solution for 30 min under a UV lamp and then treated with 5% sodium thiosulfate. Subsequently, the sections were counterstained using Nuclear Fast Red solution. Ca deposits were stained brown-black and observed under an inverted microscope (Leica, Germany).

Calcification of the MOVAS cells was observed using the Alizarin Red S staining method. Briefly, the cells were fixed in 4% formaldehyde for 5 min at 25°C, exposed to 2% Alizarin Red S (A5533, Sigma) for 30 min, and washed with 0.2% acetic acid. Calcified nodules were stained red and observed under an inverted microscope (Leica).

The Ca content in the aortic tissue section and cells were measured using the o-cresolphthalein coplexone method, as previously described [22]. Quantification of Ca (*μ*g/mg protein) was normalized to the protein concentration, which was determined using the Bicinchoninic Acid Protein Assay Kit (P0012S, Beyotime).

### 2.6. Immunofluorescence (IF) and Immunohistochemistry (IHC)

For IF analysis [23], aortic tissue sections were fixed in 4% paraformaldehyde (P0099, Beyotime) for 15 min and permeabilized with 0.3% Triton X-100 (93443, Sigma) at 25°C. The slides were stained with antibodies against LC3 (ab192890, Abcam) and P62 (ab109012, Abcam). After being washed thrice with phosphate-buffered saline (Beyotime), the membranes were probed with the corresponding secondary antibodies (Abcam). The tissue sections were then washed and incubated with 4′,6-diamidino-2-phenylindole (DAPI) staining solution (D9542, Sigma). Images were observed using a laser scanning confocal microscopy (LSCM; Leica). Then, the Image J was used to calculate green or red puncta/cell, respectively.

For IHC analysis, aortic tissue sections were fixed and subjected to immunohistochemical staining with antibody against Runx2 (ab76956, Abcam) and corresponding secondary antibody. Images were obtained using an inverted microscope (Olympus, Japan). The mean density was quantified by dividing integrated optical density (IOD) by the area of aorta using the Image Pro Plus 6.0 software.

### 2.7. GFP-mRFP-LC3B Adenoviral Vector to Detect Autophagic Flux

Autophagic flux is a typical indicator of dynamic autophagic activity, which can be evaluated using a GFP-mRFP-LC3B adenoviral vector transfection assay [24–26]. Briefly, the MOVAS cells were plated on glass-bottomed cell culture dishes (Nest, China) and infected with adenoviral vectors containing GFP-mRFP-LC3B (HanBio, China) for 24 h, according to the manufacturer's instructions. The cells were then transferred to fresh medium and incubated for 24 h. Subsequently, the cells were observed via LSCM to confirm transfection efficiency, and autophagic flux was determined by evaluating the numbers of yellow fluorescent protein (YFP) and red fluorescent protein (RFP) puncta. Yellow puncta, reflecting colocalization of RFP and green fluorescent protein (GFP) fluorescence signals, indicated the presence of autophagosomes, while free red puncta (RFP only) marked autolysosomes where acidic pH quenched GFP fluorescence.

### 2.8. Western Blot Analysis

Western blotting was performed as previously described [18]. Protein samples were separated by 8–15% SDS-PAGE and transferred to PVDF membranes (C2034, Millipore, USA). After treatment with 5% bovine serum albumin blocking reagent (SW3015, Solarbio, China) for 60 min at 25°C, the membranes were incubated overnight at 4°C with antibodies against LC3 (ab192890, Abcam), P62 (ab109012, Abcam), alpha smooth muscle actin (*α*-SMA; ab5694, Abcam), and Runx2 (ab76956, Abcam). After being washed thrice with phosphate-buffered saline (Beyotime), the membranes were probed with the corresponding horseradish peroxidase-coupled secondary antibodies (Abcam). Protein bands were visualized using a enhanced chemiluminescence (ECL) detection system (Pierce, USA) and quantified using a gel image analysis system (Bio-Rad, USA).

### 2.9. Statistical Analysis

Comparisons between multiple groups in the clinical study were analyzed using the Mann–Whitney *U* test, and correlation analysis was performed using the Spearman rank test. For the basic experiments, all values were reported as mean ± standard deviation (SD). Comparisons between multiple groups were tested by one-way analysis of variance (ANOVA), followed by Fisher's least significant difference (LSD) test. Statistical analysis was performed using IBM SPSS Statistics v19.0 software (IBM, USA). The statistical significance level was set at *P* < 0.05.

## 3. Results

### 3.1. Serum Klotho Level Is Negatively Associated with Aortic Calcification in Clinical Experiments

**Figure 1:**
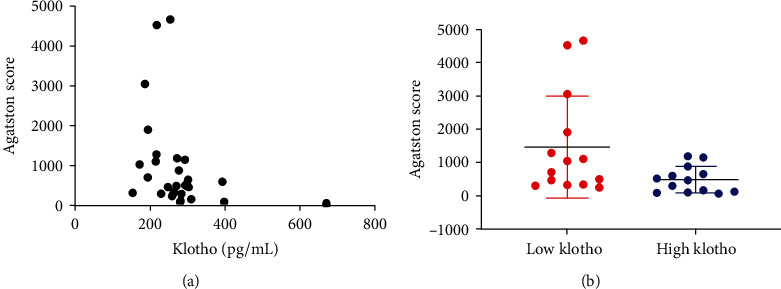
Serum Klotho level is negatively associated with calcification of aorta in clinical experiments. (a) The serum Klotho level is negatively associated with Agatston scores of aorta through Spearman rank analysis (*n* = 27). (b) the Agatston score in the low Klotho group is markedly higher than that in the Klotho group as determined by the Mann–Whitney *U* test (*n* = 27).

The spearman correlation analysis of clinical study results indicated that the serum Klotho level was negatively associated with aortic calcification, as measured by Agatston score (*P* < 0.05; Figure 1(a)). The Agatston score in the group with low Klotho serum level was significantly higher than that in the group with high Klotho serum level (*P* < 0.05; Figure 1(b)).

### 3.2. Obvious Aortic VC Appears in Klotho-Deficient Mice

**Figure 2:**
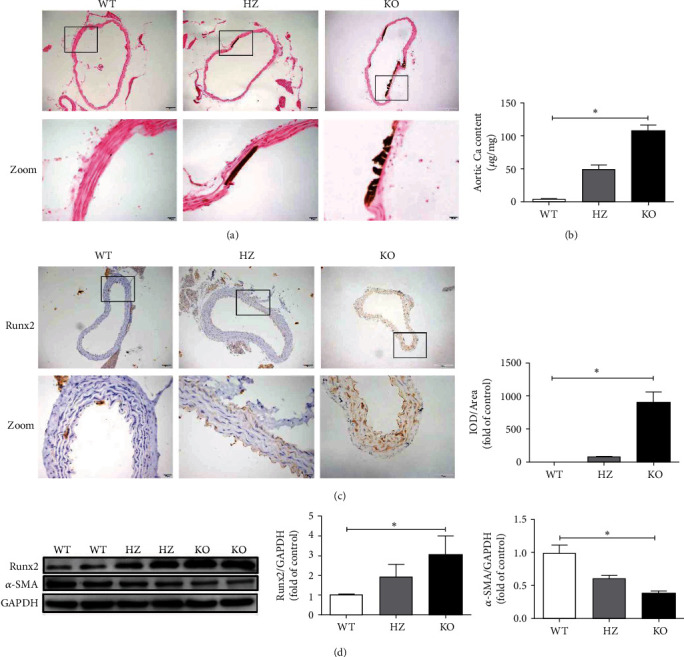
Obvious aortic VC appears in Klotho-deficient mice. (a) Klotho deficiency leads to apparent aortic calcification in Von Kossa staining experiments. (b) The Ca content of the aorta significantly increases in the KO group compared with that in the WT group (*n* = 3). (c) The expression of Runx2 significantly increases in the KO group compared with that in the WT group in IHC experiments (*n* = 3). (d) Klotho deficiency significantly increases Runx2 protein and decreases *α*-SMA protein expression in the KO group compared with those in the WT group (*n* = 3). ^∗^*P* < 0.05.

The Von Kossa staining results showed no Ca staining in the aortic tissue sections of the WT group, whereas moderate and strong positive staining were observed in the HZ and KO groups, respectively, indicating the presence of VC (Figure 2(a)). Moreover, the Ca content of the aorta increased significantly in the KO group compared with that in the WT group (*P* < 0.05; Figure 2(b)). Further, the IHC results demonstrated that aortic expression of Runx2 (a factor closely related to calcification) significantly increased in the KO group compared with that in the WT group (*P* < 0.05; Figure 2(c)). The western blot results confirmed that Klotho deficiency resulted in increased Runx2 protein expression but decreased *α*-SMA protein expression in the KO group compared with their corresponding levels in the WT group (*P* < 0.05; Figure 2(d)).

### 3.3. The Use of KL Ameliorates Aortic VC in Klotho-Deficient Mice

**Figure 3:**
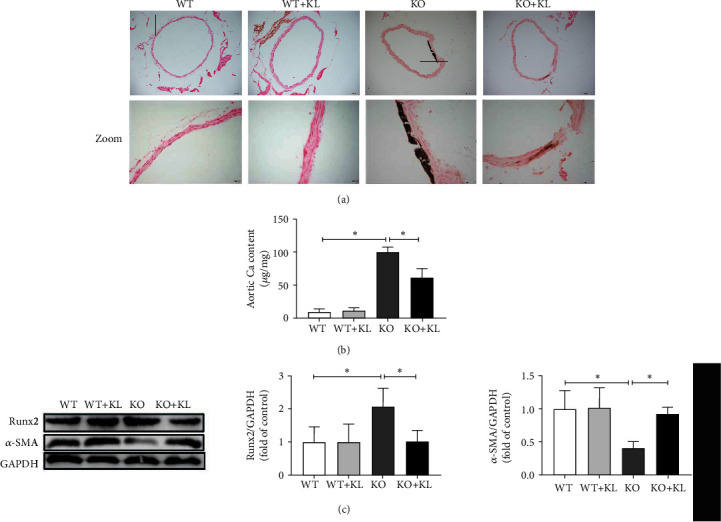
The use of KL ameliorates aortic VC in Klotho-deficient mice. (a) The Von Kossa staining results show that the use of KL relieves aortic VC. (b) The use of KL apparently reduces Ca content compared with that in the KO group (*n* = 3). (c) The expression of Runx2 protein significantly decreases and *α*-SMA protein significantly increases in the KO + KL group compared with those in the KO group (*n* = 3). ^∗^*P* < 0.05.

The Von Kossa staining results revealed that aortic Ca staining receded in the KO + KL group compared with that in the KO group (Figure 3(a)). The Ca content analysis confirmed that the Ca content of the aorta decreased significantly in the KO + KL group compared with that in the KO group (*P* < 0.05; Figure 3(b)). Further, the expression of Runx2 protein decreased significantly while that of *α*-SMA protein increased significantly in the KO + KL group compared with their corresponding levels in the KO group (*P* < 0.05; Figure 3(c)).

### 3.4. Autophagy Increases in the Aorta of Klotho-Deficient Mice

**Figure 4:**
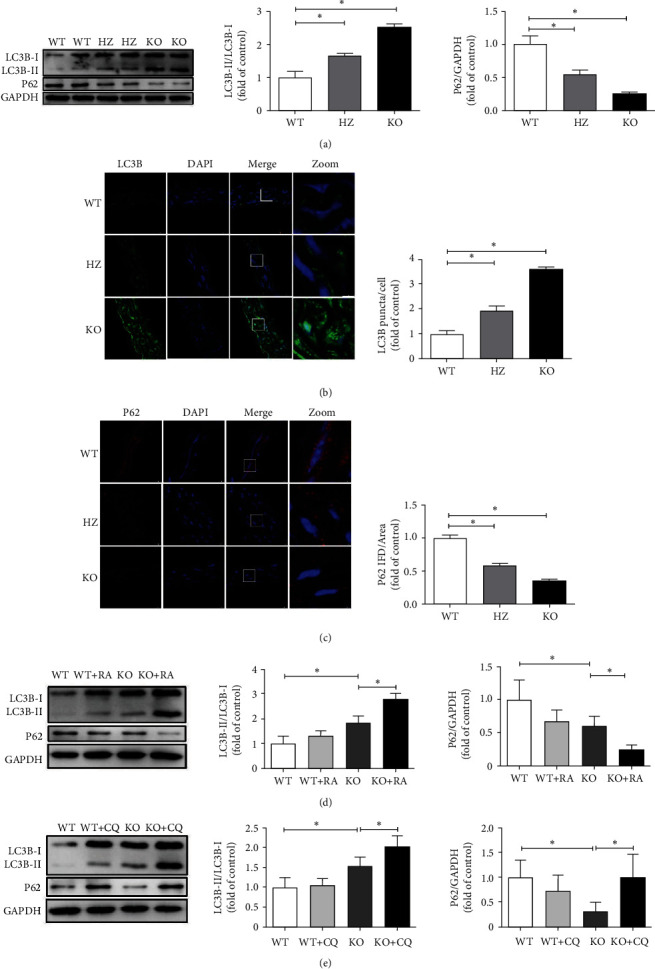
Autophagy increases in the aorta of Klotho-deficient mice. (a) Klotho deficiency induces a significant increase in LC3B-II/I ratio and decreases P62 protein expression in the KO and HZ groups compared with those in the WT group (*n* = 3). (b, c) Klotho deficiency induces a drastic increase in LC3B puncta/cell and decreases P62 fluorescence intensity in the KO and HZ groups compared with those in the WT group (*n* = 3). (d) The employment of RA significantly enhances the LC3B-II/I ratio but inhibits P62 protein expression in the KO + RA group compared with those in the KO group (*n* = 3). (e) The employment of CQ significantly increases the LC3B-II/I ratio and P62 protein expression in the KO + CQ group compared with those in the KO group (*n* = 3). ^∗^*P* < 0.05.

The western blot results revealed that the LC3B-II/I ratio considerably increased (*P* < 0.05) while P62 protein expression significantly decreased (*P* < 0.05) in the KO and HZ groups compared with their corresponding levels in the WT group (Figure 4(a)). Further, the IF results demonstrated that the number of LC3B puncta/cell significantly increased (*P* < 0.05) while the P62 fluorescence intensity markedly decreased (*P* < 0.05) in the KO and HZ groups compared with their corresponding levels in the WT group (Figures 4(b) and 4(c)). RA, an autophagy activator, and CQ, an autophagy inhibitor, were then employed to modulate autophagy. Treatment with RA markedly enhanced the LC3B-II/I ratio (*P* < 0.05) but decreased P62 protein expression (*P* < 0.05) in the KO + RA group compared with their corresponding levels in the KO group (Figures 4(d)). Conversely, treatment with CQ significantly enhanced the LC3B-II/I ratio (*P* < 0.05) and P62 protein expression (*P* < 0.05) in the KO + CQ group compared with their corresponding levels in the KO group (Figures 4(e)).

### 3.5. Autophagy Protects against Aortic VC in Klotho-Deficient Mice

**Figure 5:**
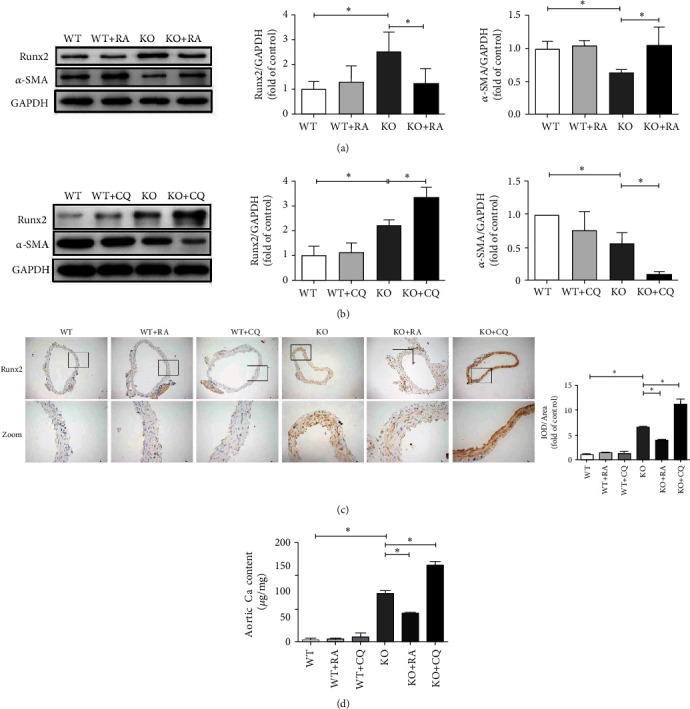
Autophagy protects against aortic VC in Klotho-deficient mice. (a) The use of RA significantly inhibits Runx2 protein expression and promotes *α*-SMA protein expression in the KO + RA group compared with those in the KO group (*n* = 3). (b) The use of CQ significantly promotes Ruxn2 protein expression and inhibits *α*-SMA protein expression in the KO + CQ group compared with those in the KO group (*n* = 3). (c) In IHC experiments, the expression of Runx2 markedly decreases in the KO + RA group and increases in the KO + CQ group compared with those in the KO group (*n* = 3). (d) The Ca content significantly decreases after the stimulation of RA but increases after the stimulation of CQ compared with those in the KO group (*n* = 3). ^∗^*P* < 0.05.

The western blot results revealed that treatment with RA significantly inhibited the expression of Runx2 protein (*P* < 0.05) but recovered *α*-SMA protein expression (*P* < 0.05) in the KO + RA group compared with their corresponding levels the KO group (Figure 5(a)). Further, treatment with CQ markedly increased Runx2 protein expression (*P* < 0.05) but decreased *α*-SMA protein expression (*P* < 0.05) in the KO + CQ group compared with their corresponding levels in the KO group (Figure 5(b)). The IHC results demonstrated a significantly decreased (*P* < 0.05) expression of Runx2 in the KO + RA group and increased (*P* < 0.05) expression of Runx2 in th KO + CQ group compared with that in the KO group (Figure 5(c)). Moreover, the Ca content results (Figure 5(d)) confirmed that RA treatment resulted in significantly reduced Ca content of the aorta in the KO + RA group (*P* < 0.05) compared with that in the KO group, whereas CQ treatment significantly enhanced the Ca content of the aorta (*P* < 0.05) compared with that in the KO group.

### 3.6. Klotho Ameliorates Aortic VC by Promoting Autophagy in Klotho-Deficient Mice

**Figure 6:**
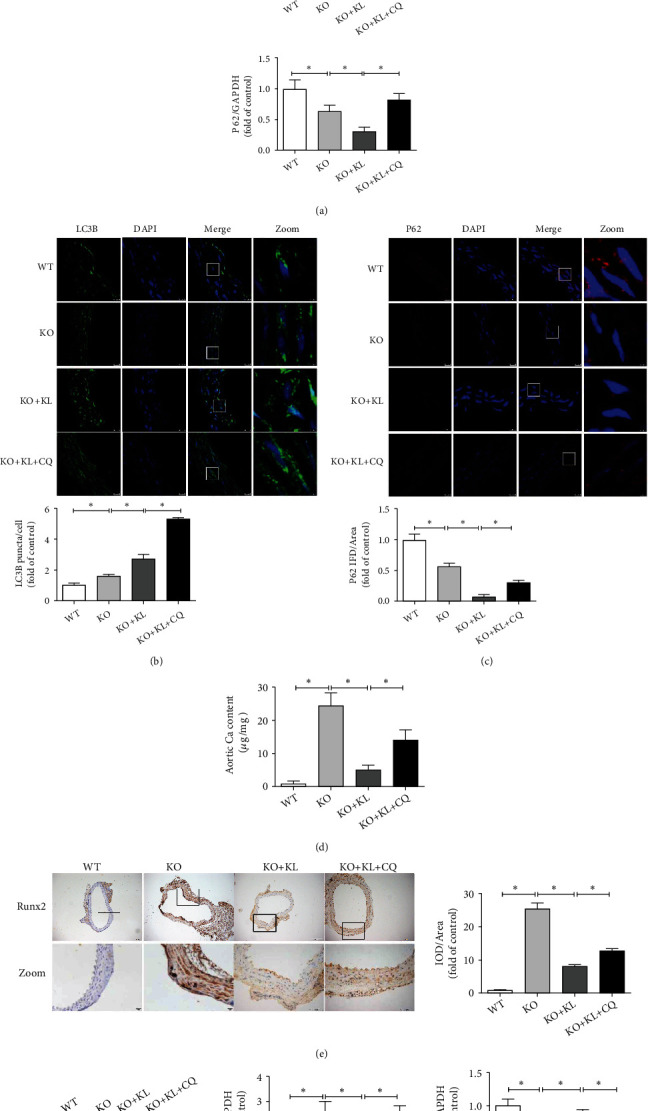
Klotho ameliorates aortic VC by promoting autophagy in Klotho-deficient mice. (a) The use of KL significantly promotes the LC3B-II/I ratio but decreases P62 protein expression in the KO + KL group compared with those in the KO group (*n* = 3). Moreover, the employment of KL + CQ further promotes the LC3B-II/I ratio but restores P62 protein expression compared with those due to treatment with KL alone (*n* = 3). (b, c) The use of KL significantly promotes LC3B puncta/cell but decreases P62 fluorescence intensity in the KO + KL group compared with those in the KO group (*n* = 3). The employment of KL + CQ further promotes LC3B puncta/cell and restores P62 fluorescence intensity compared with those due to treatment with KL alone (*n* = 3). (d) The Ca content significantly decreases in the stimulation of KL compared with that in the KO group (*n* = 3) and partially increases in the stimulation of KL + CQ compared with that due to treatment with KL alone (*n* = 3). (e) In IHC experiments, the Runx2 expression significantly decreases in the stimulation of KL compared with that in the KO group (*n* = 3) and partially recovered in the stimulation of KL + CQ compared with that due to treatment with KL alone (*n* = 3). (f) The use of KL greatly inhibits Runx2 protein expression and promotes *α*-SMA protein expression in the KO + KL group compared with those in the KO group (*n* = 3), and the employment of KL + CQ markedly increases Runx2 protein expression and inhibits *α*-SMA protein expression compared with those due to treatment with KL alone (*n* = 3). ^∗^*P* < 0.05.

The western blot results (Figure 6(a)) revealed that KL treatment significantly promoted the LC3B-II/I ratio (*P* < 0.05) but decreased P62 protein expression (*P* < 0.05) in the KO + KL group compared with their corresponding levels in the KO group. Pretreatment with CQ before KL significantly increased the LC3B-II/I ratio (*P* < 0.05) and P62 protein expression (*P* < 0.05) compared with those due to treatment with KL alone. The IF results (Figures 6(b) and 6(c)) indicated that KL treatment significantly increased the number of LC3B puncta/cell (*P* < 0.05) but decreased the P62 fluorescence intensity (*P* < 0.05) in the KO + KL group compared with their corresponding levels in the KO group. Pretreatment with CQ before KL significantly enhanced the number of LC3B puncta/cell (*P* < 0.05) and P62 fluorescence intensity (*P* < 0.05) compared with those due to KL treatment alone. These results indicated that Klotho further promoted autophagic activity *in vivo*.

Subsequently, aortic VC was evaluated when autophagy was inhibited. Pretreatment with CQ before KL significantly increased the Ca content of the aorta (*P* < 0.05) compared with that during treatment with KL alone (Figure 6(d)). The IHC results confirmed that pretreatment with CQ before KL markedly promoted aortic Runx2 expression (*P* < 0.05) compared with that due to KL treatment alone (Figure 6(e)). Moreover, the western blot results supported that pretreatment with CQ before KL markedly promoted aortic Runx2 protein expression (*P* < 0.05) and inhibited aortic *α*-SMA protein expression (*P* < 0.05) compared with that due to KL treatment alone (Figure 6(f)). These results indicated that inhibition of autophagy weakened the calcification inhibition of Klotho *in vivo*.

### 3.7. Klotho Ameliorates Calcification by Promoting Autophagy in MOVAS Cells

**Figure 7:**
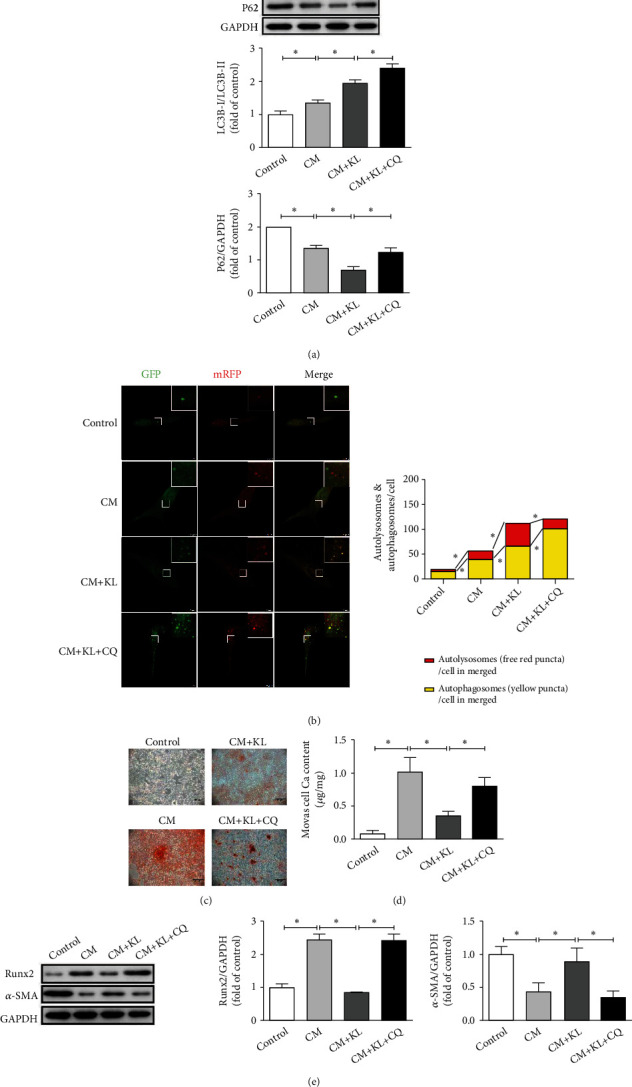
Klotho ameliorates calcification by promoting autophagy in MOVAS cells. (a) The use of KL significantly increases the LC3B-II/I ratio but decreases P62 protein expression in the CM + KL group compared with those in the CM group (*n* = 3). In addition, KL + CQ further promotes the LC3B-II/I ratio and restores P62 protein expression (*n* = 3) compared with those due to treatment with KL alone. (b) Both the number of yellow puncta and red puncta in merged images significantly increase in the CM group compared with those in the control group (*n* = 3). Moreover, the use of KL increases the number of free red and yellow puncta in merged images in the CM + KL group compared with those in the CM group (*n* = 3). The use of KL + CQ further increases the number of yellow puncta but decreases red puncta in merged images compared with those due to treatment with KL alone (*n* = 3). (c) In Alizarin Red S staining experiments, the use of KL ameliorates calcification staining in the CM + KL group compared with the CM group and the use of KL + CQ aggravates calcification staining compared with treatment with KL alone. (d) The Ca content significantly decreases after the use of KL compared with that of the CM group (*n* = 3) and markedly increases after the use of KL + CQ compared with that due to treatment with KL alone (*n* = 3). (e) The employment of KL significantly inhibits the Runx2 protein expression but promotes the *α*-SMA protein expression in the CM + KL group (*n* = 3) compared with those in the CM group, and the employment of KL + CQ significantly promotes the Runx2 protein expression and decreases the *α*-SMA protein expression (*n* = 3) compared with those due to treatment with KL alone. ^∗^*P* < 0.05.

Subsequently, the role of autophagy in the process of Klotho inhibiting calcification *in vitro* was evaluated. The MOVAS cells exhibited spindle-shaped morphology and were *α*-SMA-positive (Figure S1), verifying the properties of this cell. The western blot results (Figure 7(a)) showed that the LC3B-II/I ratio significantly increased (*P* < 0.05) and P62 protein expression significantly decreased (*P* < 0.05) in the CM group compared with their corresponding levels in the control group. Moreover, KL treatment significantly increased the LC3B-II/I ratio (*P* < 0.05) but decreased P62 protein expression (*P* < 0.05) in the CM + KL group compared with their corresponding levels in the CM group. Pretreatment with CQ before KL significantly enhanced the LC3B-II/I ratio (*P* < 0.05) and P62 protein expression (*P* < 0.05) compared with those due to treatment with KL alone.

A pH-sensitive tandem GFP-mRFP-LC3 adenoviral construct was used to detect autophagic activity of the MOVAS cells via LCSM (Figure 7(b)). The numbers of free red and yellow puncta in merged images significantly increased in the CM group (*P* < 0.05) compared with those in the control group and markedly increased after KL treatment (*P* < 0.05) compared with those in the CM group. Pretreatment with CQ before KL significantly increased the number of yellow puncta (*P* < 0.05) but decreased the number of free red puncta (*P* < 0.05) in the merged images compared with those due to treatment with KL alone. These results indicated that Klotho further promoted autophagic activity *in vitro*.

Then, cell calcification was evaluated when autophagy was inhibited. In Alizarin Red S staining experiments (Figure 7(c)), KL treatment caused less cell calcification staining in the CM + KL group compared with that in the CM group. Further, pretreatment with CQ before KL caused aggravated calcification staining compared with that due to treatment with KL alone. The Ca content analysis results (Figure 7(d)) showed that the Ca content of MOVAS cells significantly decreased (*P* < 0.05) in the CM + KL group compared with that in the CM group. Pretreatment with CQ before KL significantly increased the Ca content (*P* < 0.05) of MOVAS cells compared with that due to treatment with KL alone. Thereafter, the western blot results (Figure 7(e)) showed that Runx2 protein expression significantly increased (*P* < 0.05), and *α*-SMA protein expression significantly decreased (*P* < 0.05) in the CM group compared with their corresponding levels in the control group. KL treatment markedly inhibited cell Runx2 protein expression (*P* < 0.05) and promoted cell *α*-SMA protein expression (*P* < 0.05) in the CM + KL group compared with their corresponding levels in the CM group. Pretreatment with CQ before KL markedly promoted cell Runx2 protein expression (*P* < 0.05) and suppressed cell *α*-SMA protein expression (*P* < 0.05) compared with those due to treatment with KL alone. These results indicated that inhibition of autophagy weakened the calcification inhibition of Klotho *in vitro*.

## 4. Discussion

Our study from *in vivo* and *in vitro* experiments indicates that Klotho deficiency induces protectively increased autophagy and that Klotho expression further enhances autophagy to ameliorate calcification.

It has been previously reported that Klotho is capable of inhibiting VC [27, 28]. Both of the membrane-bound and soluble forms of Klotho can play a role. First, the combination of membrane-bound Klotho and FGF23 in the kidney leads to phosphaturia and decreased serum Pi level [29], one of the vital calcification-induced factors. Second, soluble Klotho remedies hyperphosphatemia through interaction with FGFR1 in the kidney [30]. Third, soluble Klotho may inhibit Na-Pi cotransporter activity in the VSMC membrane [31], restrain entry of Pi into cells, and inhibit osteoblastic differentiation of VSMCs. Moreover, Klotho reportedly antagonizes the Wnt/*β*-catenin pathway, reduces BMP2 expression, inhibits transcriptional activation of Runx2, and suppresses osteogenic differentiation of VSMCs [32]. Whether other mechanisms are involved remains unclear.

Autophagy, an intracellular degradation pathway, has been thought to be involved in the regulation of VC. Our results showed that high Pi increased autophagy in MOVAS cells, and inhibition of Pi-induced autophagy aggravated calcification, which is consistent with the results of a previous study [11]. Thus, autophagy may be an endogenous protective mechanism counteracting Pi-induced calcification. Augmented autophagy could interfere with the osteogenic differentiation, inhibit apoptosis and MV secretion of VSMCs, and ameliorate calcification [33]. However, there are few studies exploring the role of autophagy in Klotho deficiency-induced VC.

Klotho influences autophagy in different ways depending on the tissue and the physiological or pathological condition. Klotho usually seems to restore normal autophagy activity and treat disorders including neurodegenerative diseases and kidney diseases, suggesting the protective role of autophagy [34, 35]. As per vascular diseases, our study deems that Klotho deficiency contributes to protectively increased autophagy and VC, and added KL further enhances autophagy to ameliorate VC. However, there are some studies reporting that Klotho deficiency contributes to detrimentally increased autophagy and artery stiffening, and added KL inhibits autophagy to inhibit artery stiffening [13, 36]. These differences may be attributed to disease models, employed genotypes of mice, and age of sacrificed mice. In detail, our study employed Klotho KO mice (age 4 weeks) to establish aortic VC mice model *in vivo* and used calcification-induced MOVAS cells model *in vitro*. The aforementioned study [36] employed Klotho HZ mice (age 20-23 months) to construct aortic stiffening mice model *in vivo* and used MOVAS cells cultured in Klotho free medium model *in vitro*. Moreover, these authors [36] detected only the autophagic activity when KL was used *in vitro*. The authors should have evaluated the artery stiffening when KL was used *in vitro*, as well as autophytic activity and artery stiffening when it was used *in vivo*. Ultimately, our view on the VC inhibitory effects of Klotho through promotion of autophagy is rational.

The mechanisms of Klotho regulating autophagy attracts a lot of interest recently. We found that autophagy protectively increased both in the aorta of Klotho-deficient mice and in MOVAS cells under calcifying conditions. This may be because Klotho deficiency induces high serum Pi level, promotes the production of multiplies reactive oxygen species (ROS), and enhances autophagy, which is consistent with a previous report [37]. Subsequently, we found that added KL further increased autophagy to inhibit VC. The possible mechanisms can be summarized in the following aspects. First, Klotho inhibits IGF-1 phosphorylation, inhibits Akt and mTOR phosphorylation, and promotes autophagy [38, 39]. Moreover, Klotho can inhibit Beclin 1/Bcl2 interaction and promote autophagy [40]. Lastly, Klotho can sequester several Wnt ligands, inhibit the Wnt/*β*-catenin pathway, and promote autophagy [41–43]. Together, Klotho plays a protective function in VC, and autophagy exerts beneficial role during the process. Moreover, other potential pathways involved in Klotho regulation of autophagy warrant further investigation.

## 5. Conclusions

In conclusion, our study demonstrates that Klotho deficiency induces protectively increased autophagy and VC. Furthermore, Klotho expression may further promote autophagy to inhibit calcification. Our study provides novel fundamental evidence supporting that autophagy is one of the important mechanisms of Klotho in regulating VC, posing an important guiding significance for future clinical studies and the development of effective therapy for VC.

## Data Availability

The data used to support the findings of this study are available from the corresponding author/s on reasonable request.

## Supplementary Materials

Supplementary Materials Figure S1: IF staining of *α*-SMA in the MOVAS cells. (a) Cell nuclei are stained with DAPI (blue). (b) *α*-SMA proteins are detected in the cytoplasm of the MOVAS cells. (c) The merged image is shown.
