# Supplementary figures and images for: Klotho Ameliorates Vascular Calcification via Promoting Autophagy
Source: Oxid Med Cell Longev. 2022 Oct 26;2022:7192507. doi: 10.1155/2022/7192507 (PMC9629936; doi:10.1155/2022/7192507)

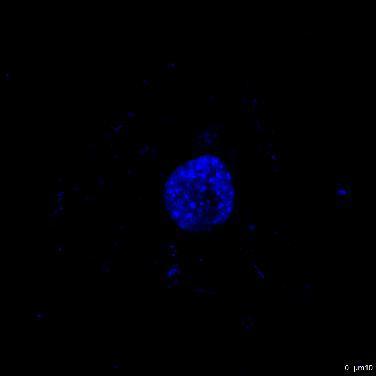

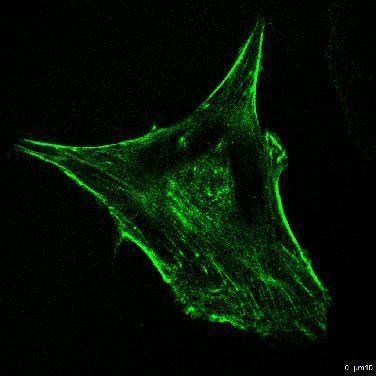

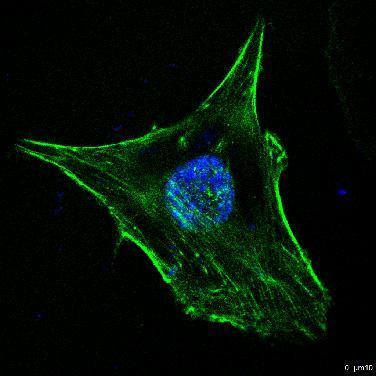


**(a) DAPI (b) α-SMA (c) Merge**

Supplement: Supplementary Materials — Figure S1: IF staining of α-SMA in the MOVAS cells. (a) Cell nuclei are stained with DAPI (blue). (b) α-SMA proteins are detected in the cytoplasm of the MOVAS cells. (c) The merged image is shown. [file 7192507.f1.docx]
